# Dynamic visual attention characteristics and their relationship to match performance in skilled basketball players

**DOI:** 10.7717/peerj.9803

**Published:** 2020-08-19

**Authors:** Peng Jin, Xiawen Li, Bin Ma, Hongbo Guo, Zhongxi Zhang, Lijuan Mao

**Affiliations:** 1 School of Physical Education and Training, Shanghai University of Sport, Shanghai, China; 2 Department of Physical Education, Nanjing University of Aeronautics and Astronautics, Nanjing, China; 3 School of Physical Kinesiology, Shanghai University of Sport, Shanghai, China; 4 College of Physical Education Science, Anshan Normal University, Anshan, China; 5 Physical Education College of Zhengzhou University, Zhengzhou, China

**Keywords:** Basketball player, Dynamic visual attention, Visual tracking speed, Match performance, Multiple object tracking

## Abstract

**Background:**

Dynamic visual attention is important in basketball because it may affect the performance of players and thus the match outcome. The goals of this study were to investigate the difference in dynamic visual attention characteristics between highly skilled basketball players and nonathletic college students and to explore the relationship between visual attention and game-related performance among the basketball players.

**Methods:**

In total, 24 highly skilled basketball players and 24 nonathletic college students participated in a multiple object tracking task. The task was conducted so that either the number of targets that were visually tracked or the speed at which a given number of tracked targets moved was altered to examine the difference in dynamic visual attention characteristics between the basketball players and nonathletic college students. The relationship between visual tracking speed (VTS) and game-related statistics, including assists, steals, mistakes, fouls and points scored recorded for every match during the season, was assessed among the basketball players by using Pearson correlations.

**Results:**

A significant main effect of target tracking load was observed (*P* < 0.001), with visual tracking performance significantly decreased as target number increased. In addition, the speed at which the targets moved had a significant effect on visual tracking performance (*P* < 0.001), with tracking performance significantly decreased as target speed increased. However, no significant difference was observed in the abilities of basketball players and nonathletic college students to simultaneously track up to six targets. By contrast, a significant interaction between group and target speed was found (*P* < 0.001), with the visual tracking accuracy of basketball players significantly greater than that of college students at the higher target speeds examined (*P* < 0.001). Among basketball players, there were positive, large, and statistically significant correlations in the accuracy in VTS trials and the number of assists (*P* < 0.001) and between the accuracy in VTS trials and the number of steals (*P* < 0.001).

**Conclusion:**

The advantage of skilled basketball players to handle dynamic visual information in a multiple object tracking task was not attributable to the target number but to the target speed. Those athletes with greater dynamic visual attention were more likely to successfully assist or to steal the ball, enhancing performance of the athlete as well as contributing to a more successful team match. These findings may inform basketball training programs to improve player and team performances during matches.

## Introduction

The ability to track multiple moving objects is important. It represents the capacity of a human brain to process multiple visual information from a dynamic scene, and it is reflected on the sports field by athletes through anticipation and decision-making skills (Marteniuk, 1976). In sports, particularly in team sports, visual attention has a critical role (Hijazi, 2013; Memmert & Furley, 2007; Memmert, Simons & Grimme, 2009). Athletes participating in team sports collect and process large amounts of visual information in a dynamic environment and must respond in a timely manner (Voss et al., 2010). Basketball is a fast-paced team sports game that requires the players to pay attention not only to the movement and position of teammates and opponents at the same time but also to the spatial position of the ball and the field (Zarić, Dopsaj & Marković, 2018). Basketball players with a good tracking ability can predict and evaluate athletic performance (Mangine et al., 2014). Therefore, studying attention processing mechanisms in skilled basketball players should provide insights into dynamic visual attention. A common paradigm used to study dynamic visual attention is called the multiple object tracking (MOT) task (Pylyshyn & Storm, 1988). In this task, observers visually track a subset of target items moving among identical distractors.

The MOT task also provides a good method to study the broader topic of continuous and dynamic visual attention (Meyerhoff, Papenmeier & Huff, 2017). A topic receiving a lot of recent research attention is comparing MOT performance of specific groups and control groups under different conditions and at various levels. For example, studies have found that air-traffic controllers show better performance on the MOT task than undergraduate students without any air-traffic training (Allen, 2004). Faubert (2013) showed that professional athletes are much better than sub-elites and novices to process complex dynamic visual scenes. Martín et al. (2017) used the MOT task and found that the tracking performance of rugby players in the back positions is better than that of those in the forward positions and better than that of undergraduate students. All of the aforementioned studies have used the MOT task to show that expertise in visual attention provides certain advantages. Individuals with such expertise can take in more complex spatiotemporal information faster than non-experts, giving the experts a greater advantage in cognitive processing of spatiotemporal information.

However, not all of the empirical evidence agrees with these results. Stothart et al. (2014) found gamers showed no better performance in multiple object tracking compare non-gamers. Memmert, Simons & Grimme (2009) showed that the expected differences in performance on the MOT task between expert and novice handball players did not appear. Grushko, Bochaver & Kasatkin (2015) found no statistical differences between controls and those more advanced in cycling sports, combat sports, or extreme sports. A potential reason for these discrepant results may be in the procedure of the multitarget tracking paradigm itself (Meyerhoff, Papenmeier & Huff, 2017). According to the “perception load theory”, the level of task perception load determines the resource allocation in attention processing (Lavie & Tsal, 1994). For basketball players and college students, the classic multitarget tracking task has a low perceptual load, and attention resources are sufficient to cope with the processing. The level of the “expert players” selected may be another key factor; lower level players may not have formed a stable and unique “expert advantage” yet. Although many studies have investigated dynamic visual attention in sports, it is still unclear what aspects of visual attention characteristics provide an advantage in expert players. Therefore, the aim of the present study was to use the MOT paradigm to further explore dynamic visual attention characteristics in basketball players.

Match performance analysis of professional sports helps coaches to understand players’ and the teams’ relevant performances to improve the training process (Sarmento et al., 2014). For basketball, there is a complex relationship between the various characteristics of match performance. Studies have found that abundant sleep (Mah et al., 2011), positive emotional state (Uphill, Groom & Jones, 2014), and better physical fitness (Fort-Vanmeerhaeghe et al., 2016) promote match performance. In addition, the ability to handle pressure at the critical moment (Wallace, Caudill & Mixon, 2013), belief in “hot hands” (Attali, 2013), imagery training, self-relaxation and self-talk (Kendall et al., 1990) all impact sports performance. Although researchers have ascertained some key factors influencing sports performance, only a few studies have explored the relationship between visual attention processing and match performance among basketball players. Chuang, Huang & Hung (2013) pointed out that attention is one of the most important factors affecting sports performance in the preparation stage. For example, before a free throw, a basketball player’s stable mood and relatively sustained attention will enhance performance. Mangine et al. (2014) assessed visual tracking speed (VTS) among National Basketball Association (NBA) players and associated their VTS with game-related measures of productivity. Possessing greater VTS may result in more positive plays as reflected by greater rates for accumulating assists and steals. Therefore, to inform basketball training practices by providing practical applications and to predict the results of competitive performance, the present study—along with the results of previous research and the fast-changing characteristics of modern basketball (Angel Gómez et al., 2008; Ibáñez et al., 2008)—also analyzed the dynamic visual attention characteristics of basketball players and assessed the correlations between these characteristics and match performance.

This study used the MOT task to assess the associations of the performance of basketball players and the characteristics of their dynamic visual information processing by changing two task conditions: tracking load and tracking speed. The two specific main objectives of this study were (1) to compare the dynamic visual attention characteristics between basketball players and nonathletic college students and (2) to evaluate the relationship between dynamic visual attention characteristics and match performance among basketball players. We hypothesized that skilled basketball players would show better visual tracking performance than nonathletic college students in the MOT task and that this advantage would enable basketball players to perform better in a game.

## Materials and Methods

### Participants

Sample size was estimated by the software G*Power3.1.9.2 (Faul et al., 2007). considering the effect size 0.90 based on the similar literature (Qiu et al., 2019), an alpha level of 0.05,the power 0.80, two tails (Cohen, 1992). It resulted in a sample size of 21 per group, to allow for dropout, we selected 24 participants per group, ending with a total of 48 participants. A total of 24 basketball players from two China University Basketball Association (CUBA) teams with more than 10 years of experience per person (mean, 10.59 years; SD, 2.39 years) from 18 to 22 years of age (mean, 20.45; SD, 2.16 years) comprised the basketball player group. All players participated the experiment. The control group was composed of 24 undergraduate students 17–21 years of age (mean, 18.73; SD, 1.64 years) with non-sport activity. All participants reported normal or corrected-to-normal vision. Each participant who completed the study was compensated for their time. The experimental protocol was approved by the regional ethics committee of the Shanghai University of Sport (No. 2017044SUS). All participants provided written informed consent prior to the start of the experiment.

### Procedure

The experiment was conducted 7–10 days after the 19th CUBA Southeast Division Tournament. We used the MOT task to explore both individual and group differences in dynamic visual attention characteristics. The task assessed two different conditions: the ability to track an increasing number of targets moving at a given speed, and the ability to track a set number of targets moving at various speeds. The tracking load task consisted of five blocks (with 2, 3, 4, 5 and 6 targets) of 30 trials each for a total of 150 trials, with a rest period of 2 min between each block. Thus, this experimental task condition lasted approximately 45 min. After a 10-min break, the tracking speed task began. This task consisted of three blocks (5°/s, 10°/s and 15°/s) of 30 trials each block for a total of 90 trials, with a rest period of 2 min between blocks. Thus, this experimental task condition lasted approximately 30 min. The order the blocks were presented was counterbalanced across participants during the two tasks.

### Stimuli and apparatus

**Figure 1:**
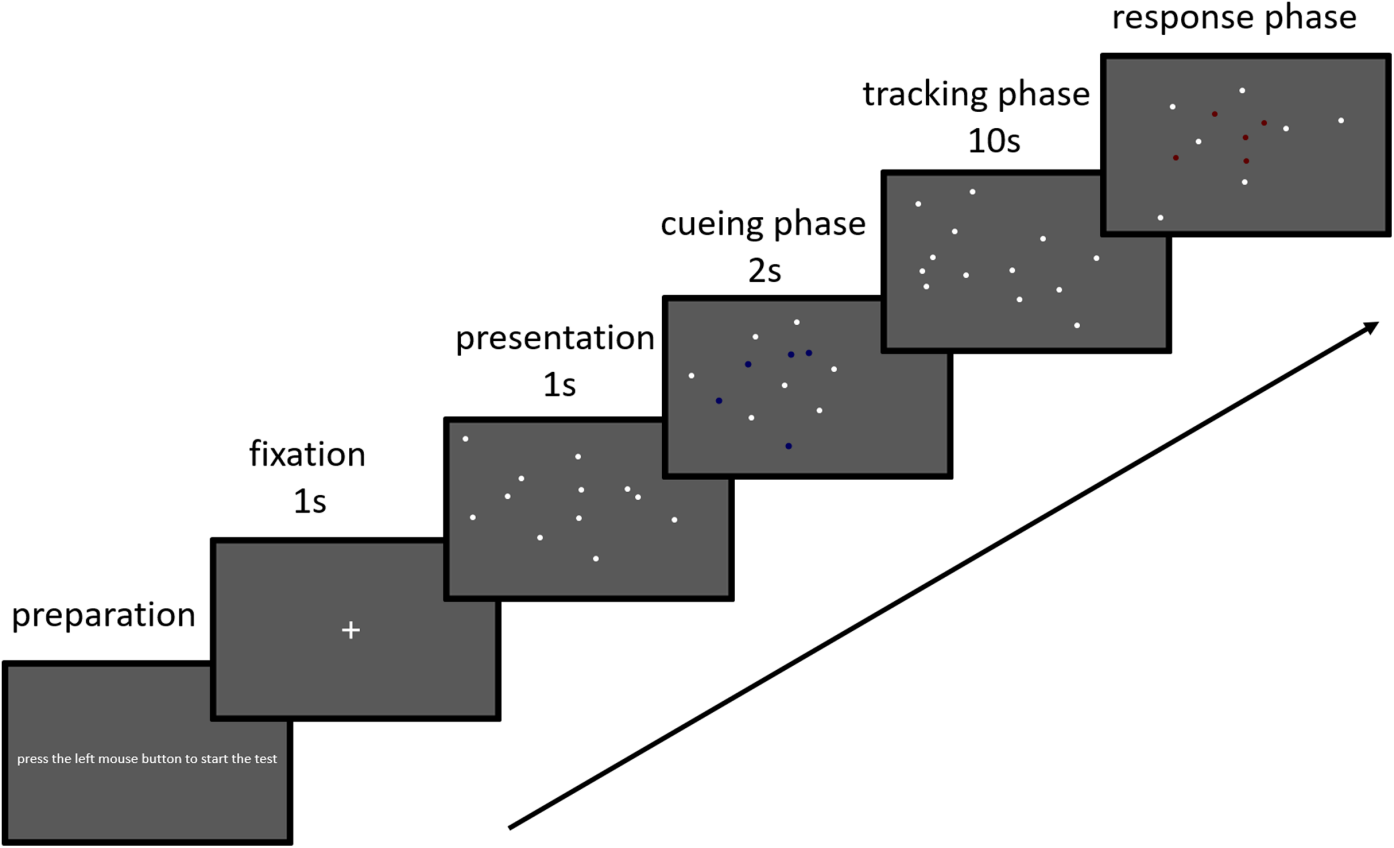
Illustration of the sequential phases included in a trial of the MOT task. Twelve white circular dots were displayed. Then a subset of the dots flashed on and flickered three times for 2 s, indicating that they were the targets for that trial. All 12 dots then moved for 10 s. At the end of the tracking period, the dots became stationary, and the participant had to click using a mouse over the target dots.

The experiment was conducted using a Dell Inspiron laptop with MATLAB 2016a and Psychtoolbox 3. Visual stimuli were displayed on a 15.6-inch monitor. The monitor had 1,920 × 1,080 pixels and a refresh rate of 60 Hz. Participants were seated approximately 50 cm from the computer screen in a quiet room. At the beginning of the experiment, the preparation screen was presented (press the left mouse button to start the test). The preparation screen was presented in the beginning of each block only. Then a white fixation cross (+) was presented first for 1,000 ms on a gray background (37.98° × 21.0°), followed by the presentation of 12 white circular dots (diameter 0.65°) for 1,000 ms. In the tracking load task, a subset of the dots (2, 3, 4, 5, or 6) were highlighted in blue and flickered three times for 2 s to designate them as the targets. Next, all 12 dots moved in random directions at a constant speed (5°/s). They changed their directions randomly and when a dot reached the edge of the screen border. There were no additional constraints in the dots’ trajectories so there was the possibility that they occluded one another for an instant. In the tracking speed task, three dots were highlighted in blue and flickered three times for 2 s to designate them as targets for this task. Next, all 12 dots moved in random directions at a speed of 5°/s, 10°/s, or 15°/s, After 10 s, the dots stopped moving. Pressing a mouse button was required to point out the targets and to then start the next trial (Fig. 1).

### Statistical analysis

The data were recorded and collected by MATLAB 2016a software. SPSS, version 21.0, software was used to calculate the accuracy and to conduct statistical analyses. In the tracking load experiment, a mixed experimental design of 2 (group) × 5 (target number) was used. A Linear mixed effect model (LMM) was used to assess the impact of the group (basketball players, nonathletic college students), tracking load (2, 3, 4, 5 and 6 targets) and the group-by-load interaction. In the tracking speed experiment, a mixed experimental design of 2 (group) × 3 (target speed) was used. A LMM was used to assess the impact of the group (basketball players, nonathletic college students), tracking speed (5/s, 10°/s and 15°/s) and the group-by-speed interaction. Using a random intercept to account for the repeated measures of each participant. Further analyses were conducted using the simple effects test for any significant interaction. Pearson’s correlation coefficient was used to test the linear relation between visual attention and game-related performance. An alpha level of 0.05 was pre-selected for all statistical comparisons. The accuracy rate was calculated by determining the percentage of correctly selected targets across all experimental times at different target numbers for each participant.

### Match-related performance statistical analysis

All performance statistical data were obtained from the official website of China University Basketball League. Five statistics (seven games in total) were selected for match performance, including points scored, steals, assists, turnovers and fouls. Over the course of the season, the plays average 219.04 ± 32.13 minutes played. To normalize the data for individual differences in playing time, these statistics were analyzed per 100 min played. The standardized data were 7.32 ± 3.32 assists per 100 min, 4.67 ± 2.07 steals per 100 min, 8.31 ± 3.38 turnovers per 100 min, 9.68 ± 3.79 fouls per 100 min and 34.01 ± 12.13 points scored per 100 min.

## Results

### Visual tracking load assessment

**Figure 2:**
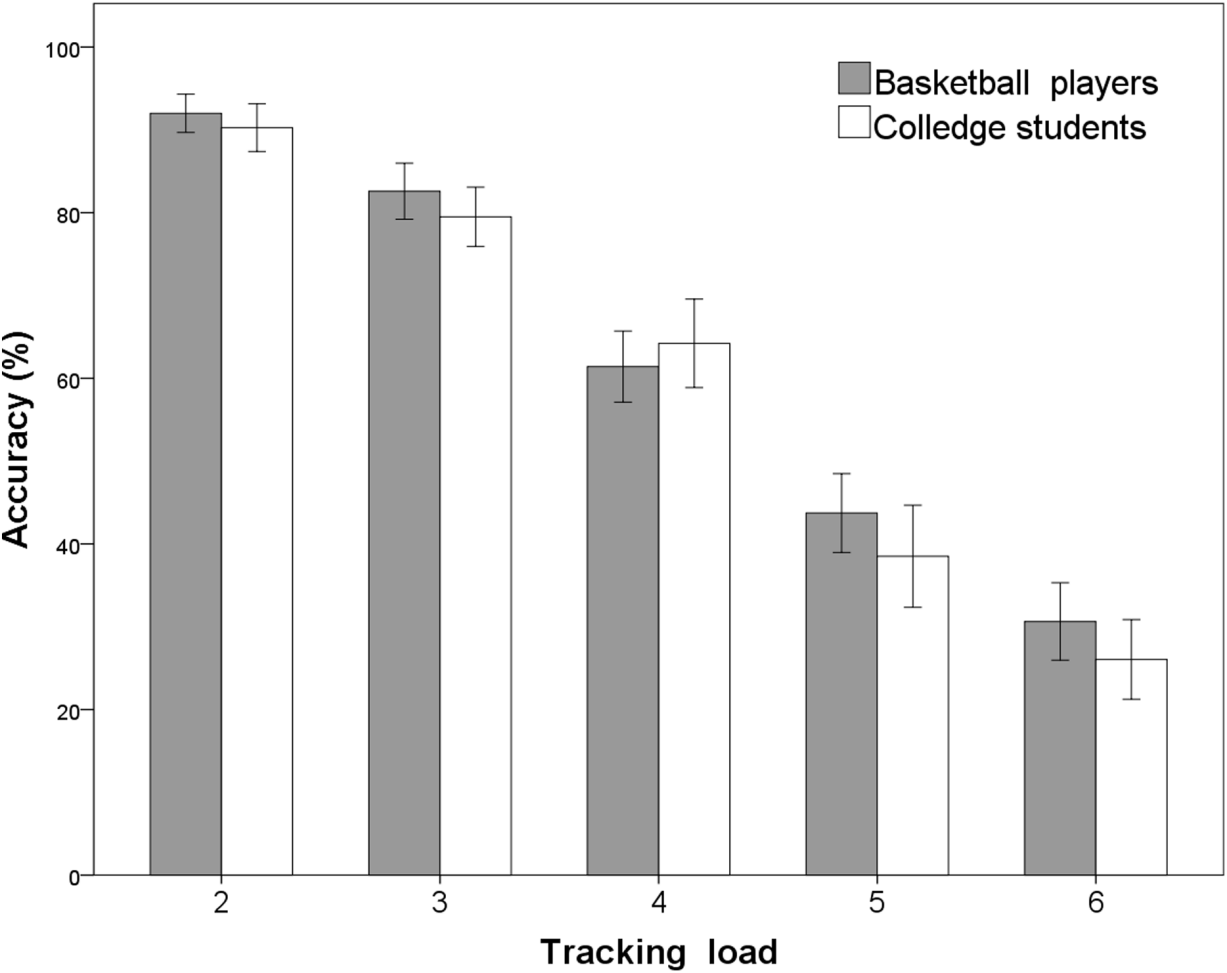
Tracking accuracies with an increasing number of targets in skilled basketball players and in nonathletic college students. It shows that the results of two groups of participants under different tracking target numbers.

Linear mixed effect model revealed a significant main effect of target load (*F* = 1371.842, 95% CI [−0.17 to −0.15], *P* < 0.001), indicating that tracking performance decreased significantly as target number increased. Tracking accuracy also significantly decreased with increasing target number (2 vs. 3, mean difference (MD) = 0.126, 95% CI [0.08–0.17], *P* < 0.001, 3 vs. 4, MD = 0.181, 95% CI [0.12–0.24], *P* < 0.001; 4 vs. 5, MD = 0.189, 95% CI [0.12–0.26], *P* < 0.001; 5 vs. 6, MD = 0.128, 95% CI [0.07–0.19], *P* < 0.001). However, there was no significant main effect of group (*F* = 1.649, 95% CI [−0.10 to −0.02], *P* = 0.204). There was no significant interaction between the group and target load (*F* = 1,371.842, 95% CI [−0.01 to −0.03], *P* = 0.256) (see Fig. 2). Thus, these results did not support our hypothesis that basketball players would have better tracking performance than nonathletic college students when the number of targets increased (up to six). This lack of a significant effect indicated that basketball players and nonathletic college students have the same level of accuracy for simultaneously tracking at least six objects.

### VTS assessment

**Figure 3:**
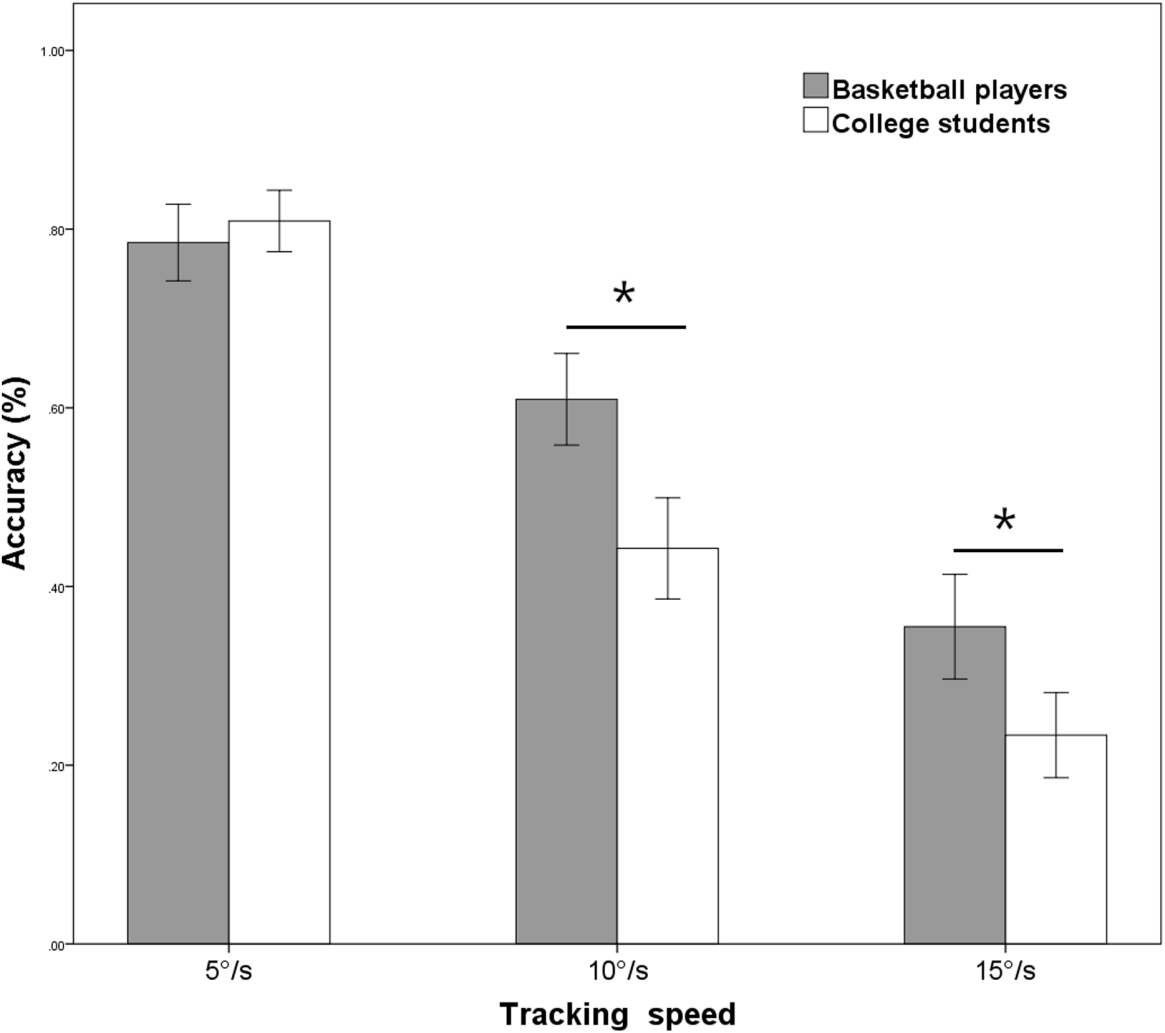
Tracking accuracies at different target speeds stratified by group. It shows that the results of two groups of participants under different tracking target speeds. *P < 0.05.

Linear mixed effect model revealed a significant interaction between the group and target speed (*F* = 19.346, 95% CI [0.04–0.11], *P* < 0.001). The results of a simple effects test showed that when the target speed was 5°/s, there was no significant difference in visual tracking accuracy between basketball players and college students (*F* = 0.339, 95% CI [−0.10 to 0.05], *P* = 0.456). However, when the target speed was 10°/s, there was a significant difference in visual tracking accuracy between basketball players and nonathletic college students (*F* = 24.731, 95% CI [0.10–0.24], *P* < 0.001), the accuracy of basketball players was significantly greater than that of nonathletic college students; when the target speed was 15°/s, there was a significant difference in visual tracking accuracy between basketball players and nonathletic college students (*F* = 13.763, 95% CI [0.10–0.24], *P* < 0.001), the accuracy of basketball players was significantly greater than that of nonathletic college students (see Fig. 3). Consistent with our hypothesis, the results of the tracking speed task indicated that with increasing target speed, basketball players had a significantly greater ability to visually track targets compared with nonathletic college students.

### Relationship between accuracy of visual tracking speed trials and match performance statistics

Pearson correlations between the accuracy of visual tracking speed trials (AVTS) and match statistics were calculated. (The magnitude of each correlation was assessed using the following scale: <0.1 = trivial; 0.1–0.3 = small; 0.3–0.5 = moderate; and 0.5–0.7 = large) (Hopkins et al., 2009). The analyses revealed that for AVTST at 10°/s and at 15°/s there was a positive, large, and statistically significant correlation between the AVTS and the number of assists (for 10°/s: *r* = 0.557, *P* = 0.005; for 15°/s *r* = 0.621, *P* = 0.001), suggesting that greater AVTS was significantly correlated with a higher number of assists (Table 1). In addition, the number of steals also had a positive, large and statistically significant correlation at the same tracking speeds (for 10°/s: *r* = 0.600, *P* = 0.002; for 15°/s: *r* = 0.687, *P* < 0.001), indicating that greater AVTS was correlated with a higher number of steals. A positive, small correlation was found between the AVTS at all three target speeds and the number of turnovers (for 5°/s: *r* = 0.257, *P* = 0.225; for 10°/s: *r* = 0.113, *P* = 0.599; for 15°/s: *r* = 0.269, *P* = 0.203). However, no significant relationships were observed between the AVTS at 5°/s and the match performance statistics of assists (*r* = 0.273, *P* = 0.195), steals (*r* = 0.345, *P* = 0.099), fouls (*r* = −0.01, *P* = 0.997), or points scored (*r* = 0.126, *P* = 0.559); no significant relationships were observed between the AVTS at 10°/s and the match performance statistics of fouls (*r* = −0.119, *P* = 0.351) and points scored (*r* = 0.227, *P* = 0.287); no significant relationships were observed between the AVTS at 15°/s and the match performance statistics of fouls (*r* = 0.178, *P* = 0.405) and points scored(*r* = 0.105, *P* = 0.625).

**Table 1:** Correlation between accuracy of visual tracking speed and match performance statistics in basketball players.

Target speed	Assists	Steals	Turnovers	Fouls	Points scored
5°/s					
Pearson *r* coefficient	0.273	0.345	0.257	−0.001	0.126
*P* value	0.197	0.099	0.225	0.997	0.559
10°/s					
Pearson *r* coefficient	0.557**	0.600**	0.113	−0.199	0.227
*P* value	0.005	0.002	0.599	0.351	0.287
15°/s					
Pearson *r* coefficient	0.621**	0.687**	0.269	0.178	0.105
*P* value	0.001	0.000	0.203	0.405	0.625

**Note:**

** *P* < 0.01.

## Discussion

The aims of the present study were to examine the difference in dynamic visual attention characteristics between basketball players and nonathletic college students as well as the relationship between dynamic visual attention characteristics and match performance among basketball players. Somewhat surprisingly, there was no significant difference between basketball players and nonathletic college students in the tracking number task. That is, both groups were equally proficient at simultaneously tracking up to six targets, with all targets moving at the same speed. By contrast, a significant difference in the tracking speed task was found between basketball players and nonathletic college students. The expertise of skilled basketball players provided them with an advantage for tracking targets moving at higher speeds. Faster tracking speed was also significantly correlated with the match performance statistics of assists and steals among basketball players. Our results suggest that a basketball player with greater dynamic visual attention is more likely to contribute to more positive plays, thereby promoting individual and team performances.

### Visual tracking performance for the number of targets

The results of the tracking number task showed that as the number of targets visually tracked increased, the accuracy in tracking the targets decreased for all participants. There was a significant main effect of target number, such that tracking accuracies were markedly better when participants had to track only two targets compared with when they had to keep track of three to six targets. The flexible-resource model suggests that the capacity of attention resources is limited: with an increase in the number of targets, fewer resources are allocated to each target, which leads to a decrease in tracking accuracy (Alvarez & Franconeri, 2007). We found no difference in the tracking performance between skilled basketball players and nonathletic college students on the MOT task. These results are inconsistent with those observed in another study using a MOT task that found that team ball players have better visual attention than non-athletes (Qiu et al., 2018). These discrepant results are likely attributable to the target speed being too slow to assess this skill for both skilled basketball players and nonathletic college students. The objects moving at a speed of 10°/s in the Qiu’s study, but the objects moving at a speed of 5°/s in our experiment. That is, the attention resources of the participants in both groups were adequate for dealing with the level of load difficulty in the present study; Pylyshyn (2004) has shown that people can typically track four to five targets and maintain a good level of accuracy. A second potential reason for our findings is that because the targets in the MOT task have no special characteristics, the results cannot reflect the attention advantage of athletes in the process of a skilled operation. Memmert, Simons & Grimme (2009) stated that experts and novices do not differ on any basic abilities, and that all the differences observed are attributable to domain-specific or sport-specific advantages. Chase & Simon (1973) found that chess players do not have a better memory for pieces on the board in general, they only exhibit such an advantage when they form a meaningful chess pattern. The work by Furley & Memmert (2010) also support this view. Visual attention is composed of working memory storage capacity and visual information processing speed (Wiegand et al., 2013). In the classic MOT task, work memory storage capacity may be assessed more than visual attention. The discrepancy between studies that have indicated that athletes are faster than non-athletes at processing visual information suggests that using target speed as a characterization of individual differences in the ability to track targets may be a better measure of visual attention in the MOT task.

### Visual tracking performance associated with target speed

Consistent with previous research (Huff et al., 2010; Tombu & Seiffert, 2011), all participants in the present study showed that visual tracking ability declines as target speed increases in the MOT task (the main effect of tracking target speed was statistically significant). When the speed increased, it always goes along with increasing the number of close encounters between target and distractors, the *close encounters model* indicates that the reduction in tracking performance as speed increases is due to the increased number of close encounters (Franconeri, Jonathan & Scimeca, 2010); due to accelerations, the target/distractors crossed more often with high speed than with low speed (Bae & Flombaum, 2012). Meyerhoff et al. (2016) showed that increasing object speed, which in return increases average crowding, more dynamically changing inter-object spacing provide the major source for tracking errors. On other hand, with the acceleration of the target, accuracy was gradually reduced. This is likely because the attention resources required by each target tracked gradually increased, while the total amount of attention resources possessed by each participant remained unchanged; thus, the participants could not effectively track the moving targets (Furley & Memmert, 2010). Of primary interest to us was the interaction between the group and target speed indicating whether basketball players differed from nonathletic college students. Our results showed that the accuracy of basketball players in tracking the targets was significantly greater than that of nonathletic college students at target speeds of 10°/s and 15°/s. This finding indicates that basketball players have a marked advantage over nonathletic college students in attention to processing speed in multitarget tracking tasks. Martín et al. (2017) investigated attention between rugby players who played different positions in the game and non-athletes by adjusting the target speed in the MOT task. Their results showed that the visual tracking performance of rugby players in defender positions is significantly better than that of both players in forward positions and in non-athletes. When the ability of NBA players at different positions in the game to track multiple targets was investigated, the result showed that the target tracking speed of guards was significantly better than that of forwards and the center and that target tracking speed ability can predict and evaluate the performance of a match (Mangine et al., 2014).

Many literature indicated that sport training improves visual attention performance, (Qiu et al., 2018; Heppe et al., 2016; Verburgh et al., 2014). In most sports, athletes must complete the established technical actions as rapidly as possible (capture effective information in a short period of time) and as accurately as possible (showing a strong spatial positioning ability). Basketball requires the players to have a strong ability to process the visual information of fast movement to make judgments and decisions within a short time. Therefore, the ability to track fast targets is important for basketball players, and this skill is in line with the requirements of the sport. Thus, expertise in this sport is related to high levels of performance on measures of processing speed and visual attention. The three-dimensional MOT speed thresholds of soccer players have been shown to be qualitatively similar to those previously obtained in professional and elite amateur athletes showing superior capacity for processing a complex and dynamic visual scene task (Romeas, Guldner & Faubert, 2016). According to the previous work, there are sudden direction changes which challenge the visual system (Horowitz & Cohen, 2010), unpredictable change of the motion direction of targets is sufficient to impair tracking performance (Meyerhoff et al., 2013). Basketball involves not only monitoring the speed and direction of the ball but also tracking the positions of teammates and opponents on the court. These tracking processes are very similar to those in the MOT task. Our results support the hypothesis that a basis for the transfer of the basketball players’ skills is that training and transfer tasks recruit overlapping cognitive processes (Dahlin et al., 2008; Jonides, 2004).

### The relationship between tracking speed and match performance

It is widely accepted that expertise in sports is related to high levels of performance on measures of processing speed and visual attention (Alves et al., 2013; Faubert, 2013; Faubert & Sidebottom, 2012). But there is a dearth of research investigating the relationship between the visual attention of basketball players in the MOT task and their basketball match performance. The present study found several relationships between measures of visual attention and match performance variables. Mangine et al. (2014) examined the assessment of AVTS in NBA players and game-related measures of productivity. Consistent with our findings, their results indicated that greater AVTS may contribute to more positive plays as reflected by greater rates of assists, steals, and turnovers. In another team ball sport, Poltavski & Biberdorf (2015) showed that faster response time and recognition speed of visual stimulation and a better visual shifting ability are significantly related to sports performance among collegiate hockey players. The results of the present study showed that greater AVTS is highly and positively correlated with the number of assists, indicating that athletes with good dynamic visual attention will have more assists in competition. In basketball, players monitor the movements and positions of several players (teammates and opponents) to make good passes rapidly, which is similar to tracking multiple objects simultaneously in the MOT task. Players with fast visual information processing will make more assists. Similarly, we found that steals also had a positive, large, and statistically significant correlation with greater tracking speed in the MOT task. In a basketball match, the skill and ability to steal the ball are signs of a quality defense. A player in a defense position has to rapidly judge and analyze key visual information to make an interception in time. Previous research has suggested that experienced basketball players reflect better visual perceptual processing to extract critical information in basketball than other sports-specific skills (Millslagle, 2002). However, good visual attention is the basis for and execution of a steal. The present study did not detect any other relationships between AVTS and the other game-related statistics examined.

Our findings supported our hypotheses in part. Basketball players with good dynamic visual attention characteristics were likely to contribute to more assists and steals. However, individual assist and steal performances are affected by many factors, including core stability and physical agility (Mcgill, Andersen & Horne, 2012). Thus, a direct one to one relationship is not expected. Nevertheless, our results do suggest that good visual attention in complex and unpredictable dynamic contexts is a critical component during match performance of elite athletes.

### Limitations

Study limitations to the present study to be pointed. It is just a correlational study, not a Randomized Controlled trial. It is impossible for our paradigm to assign subjects randomly between the conditions, it is always possible that basketball players are not better in tracking faster objects, but that people who were good at tracking fast objects continued playing basketball. Second, previous studies suggested that different positions have different visual attention characteristics because of their responsibilities on the basketball court, future research can explore the relationship between visual attention characteristics and sports performance in different positions.

## Conclusions

Our hypotheses that skilled basketball players would show better visual tracking performance than nonathletic college students in the MOT task and that this advantage would enable basketball players to perform better during a basketball match were supported in part by the results of our study. Compared with nonathletic college students, basketball players had superior high-speed visual target tracking accuracy although no significant difference was found between basketball players and nonathletic college students in the number of targets they could accurately track at a given speed in the MOT task. The high-speed visual target tracking accuracy of basketball players was also positively correlated with the number of assists and steals they successfully executed during real-world basketball matches, suggesting that visual target tracking accuracy is associated with improved game performance among skilled basketball players.

## Supplemental Information

10.7717/peerj.9803/supp-1 Supplemental Information 1 Match-related performance statistics. All performance statistical data were obtained from the official website of China University Basketball League. Five statistics were selected for match performance, including points scored, steals, assists, turnovers, and fouls.

10.7717/peerj.9803/supp-2 Supplemental Information 2 The tracking accuracy of each participant (basketball players and college students) under different target number conditions. The data includes tracking for 2 targets, 3 targets, 4 targets, 5 targets and 6 targets.

10.7717/peerj.9803/supp-3 Supplemental Information 3 The tracking accuracy of each participant (basketball players and college students) under different target speed condition. The data includes tracking 2 targets, 3 targets, 4 targets, 5 targets and 6 targets.

## Additional Information and Declarations

### Data Availability

The following information was supplied regarding data availability: The raw measurements are available in the Supplemental Files.
